# Circadian Variations in Cognitive Performance Among Patients With Narcolepsy and Obstructive Sleep Apnea: A Comparative Study

**DOI:** 10.31083/AP48699

**Published:** 2025-12-01

**Authors:** Yihong Cheng, Ruichen Fang, Leqin Fang, Dhirendra Paudel, Yan Xu, Aike Wu, Shengpeng Liang, Jinnong Jiang, Yuling Wang, Dan Zhou, Bin Zhang

**Affiliations:** ^1^Department of Psychiatry, Sleep Medicine Center, Nanfang Hospital, Southern Medical University, 510515 Guangzhou, Guangdong, China; ^2^Key Laboratory of Mental Health of the Ministry of Education, Southern Medical University, 510515 Guangzhou, Guangdong, China

**Keywords:** narcolepsy, circadian variations, cognitive performance, excessive daytime sleepiness

## Abstract

**Background:**

Patients with narcolepsy experience excessive daytime sleepiness (EDS) and cognitive impairment. However, studies on the circadian variability associated with cognitive impairment in narcolepsy patients are scarce. This study aimed to explore circadian cognitive performance in narcolepsy patients compared with patients with obstructive sleep apnea (OSA) and EDS (OSA-with-EDS).

**Methods:**

A total of 62 participants, 29 with narcolepsy and 33 with OSA-with-EDS completed the study. The assessments were done using questionnaires, polysomnography (PSG), the multiple sleep latency test (MSLT), and cognitive-behavioral tasks at different time points (20:00, 08:00, 10:00, 12:00, 14:00, 16:00, and 18:00) including the psychomotor vigilance task (PVT), the Stroop color-word task, and the 2-back task to separately assess the circadian variations of vigilant attention, inhibitory control, and working memory respectively.

**Results:**

Narcolepsy patients showed significant within-subject circadian variations in vigilant attention (*p* < 0.001), inhibitory control (*p* = 0.016), and working memory (*p* < 0.001) in the time domain. Overall, vigilant attention in narcolepsy patients presented a pattern with optimal performance observed at 20:00 on the previous night followed by deterioration in the morning (08:00~14:00) and improvement in the afternoon (14:00~18:00). Inhibitory control displayed a pattern of “enhancement in the morning (08:00~12:00) followed by a decline in the afternoon (12:00~18:00)”, while working memory displayed a trend of improvement during daytime hours, with these two measures showing their poorest performance at 20:00 on the previous night.

**Conclusions:**

Circadian variations were prominently observed in vigilant attention, inhibitory control, and working memory performance among patients with narcolepsy. Except for EDS, the intrinsic disease specificity may play an important role in the cognitive impairments associated with narcolepsy.

## Main Points

1. The cognitive function of patients with narcolepsy shows rhythmic changes during the day.

2. Compared with obstructive sleep apnea (OSA)-with-excessive daytime sleepiness (EDS) patients, patients with narcolepsy performed poorly in vigilance attention tasks, but performed better in inhibitory control and working memory tasks.

3. Intrinsic disease specificity plays an important role in the cognitive impairments associated with narcolepsy.

## 1. Introduction

Narcolepsy is a central disorder characterized by hypersomnolence, resulting from the loss or dysfunction of hypothalamic neurons that regulate the neuropeptide hypocretin [1]. This chronic neurological condition affects approximately 0.025% to 0.05% of the general population [2]. Furthermore, almost half of these people experience cognitive impairments, which are the second most prominent symptoms after excessive daytime sleepiness (EDS) impacting their daily functioning [3]. Patients with narcolepsy frequently report cognitive impairments across distinct domains of attention, executive functions, and memory [4]. These impairments typically impose a lifelong burden that adversely affects their quality of life, productivity, as well as educational and employment outcomes [5].

Research has demonstrated that cognitive function exhibits a circadian rhythm [6], typically peaking in the morning, declining in the afternoon, and recovering during the evening [7]. Additionally, cognitive performance among patients with narcolepsy varies at different time points [8]. For instance, vigilant attention is notably poorer immediately after awakening compared with the previous night but shows improvement by late morning [9]. Inhibition control in narcoleptic patients tends to be most impaired 1–2 hours after waking [10]. An early investigation into the temporal course of attention in narcoleptic patients showed a distinct U-shaped pattern [8]. However, previous studies have primarily focused on limited time points and have been unable to comprehensively delineate various aspects of cognitive function among patients with narcolepsy. Consequently, it is imperative to investigate the circadian variations in cognitive impairment within this population to provide a scientific basis for patients to arrange their study and rest reasonably.

Previous studies have predominantly compared individuals with narcolepsy with healthy controls [9, 11]. To date, there has been little research comparing patients across different diagnostic categories of sleep disorders that share similar symptoms of EDS [8]. It is known that obstructive sleep apnea (OSA) is associated with EDS [8, 12]. Using OSA-with-EDS patients as a control group can mitigate the impact of EDS and yield valuable insights into the characteristics of cognitive impairment in narcolepsy patients.

Therefore, this study aimed to investigate the circadian variability of vigilant attention, inhibitory control, and working memory through multiple time point cognitive assessments among patients with narcolepsy and compare that with OSA-with-EDS patients.

## 2. Methods

### 2.1 Participants

Participants were patients who initially visited the Sleep Medicine Center of Nanfang Hospital, Southern Medical University due to EDS from 2023 to 2024. They all underwent polysomnography (PSG) and multiple sleep latency tests (MSLT).

A multi-observation repeated measurement comparative design was used in this investigation. A total of 29 patients with narcolepsy (18 diagnosed with type 1 and 11 diagnosed with type 2) and 33 OSA-with-EDS patients were included in the analysis. The inclusion and exclusion criteria for the two groups were as follows.

Inclusion criteria for the narcolepsy group: (A) Participants meeting the diagnostic criteria of the International Classification of Sleep Disorders, Third Edition (ICSD-3) for narcolepsy [13]; (B) Participants with the capacity to comprehend and adhere to the research protocol; (C) Provide written informed consent prior to participation.

Exclusion criteria for the narcolepsy group: (A) Patients with unresolved physical or mental disorders; (B) Shift workers, including night shift employees, and frequent cross-time zone travelers (such as international flight crews); (C) Pregnant or lactating women; (D) Any additional circumstances that render an individual unsuitable for study participation.

The diagnostic inclusion criteria for the OSA-with-EDS group were Apnea-Hypopnea Index (AHI) ≥5, Epworth Sleepiness Scale (ESS) score >10, and without narcolepsy [14]. Other inclusion and exclusion criteria were similar to those established for the narcolepsy group.

### 2.2 Measurement

#### 2.2.1 Polysomnography and Multiple Sleep Latency Test

PSG recordings were performed according to the American Academy of Sleep Medicine (AASM) Manual for the Scoring of Sleep and Associated Events guidelines [15]. Nocturnal PSG recording was conducted starting at 22:00 and ending at 07:00 in this study.

All participants underwent the MSLT following the nocturnal PSG monitoring according to the AASM Practice Parameters for Clinical Use of the MSLT [15]. The test included five 20-minute naps spaced 2 hours apart, starting at 09:00. Both PSG and MSLT recordings were scored manually by sleep specialists.

#### 2.2.2 Questionnaires 

The sleep and psychological states of the participants were assessed through self-rating scales.

EDS was assessed using the ESS, an eight-item patient-reported outcome measure to assess the likelihood of falling asleep under various conditions. Each item scored from 0 (would never doze) to 3 (high chance of dozing), with a total score ranging from 0 to 24. A score >10 was used as the threshold for clinically significant EDS. The scale demonstrated good reliability, with a Cronbach’s alpha of 0.814, supporting its validity for this assessment [16].

The participants’ subjective sleep quality was evaluated by the Pittsburgh Sleep Quality Index (PSQI), a validated instrument comprising seven components. Each component was scored on a scale from 0–3, with the total score ranging from 0–21; where a higher score describes poorer sleep quality. A total PSQI score greater than 5 has been validated as being highly sensitive and specific in distinguishing good from poor sleepers across several populations. Study have shown that Cronbach’s alpha for each item of the PSQI was 0.875 [17].

Chronotype was assessed using the Morningness-Eveningness Questionaire-5 (MEQ-5), which contained five items. The total score ranged from 4 to 25, where 18 to 25 points were defined as the morning chronotypes, 12 to 17 points were defined as the neutral chronotypes, and 4 to 11 points were defined as the evening chronotypes. Chronotype was examined in this study as a continuous score, with lower scores indicating greater eveningness. Cronbach’s alpha coefficient in this study was 0.74 [18].

Anxiety and depression symptoms were assessed using the Hospital Anxiety and Depression Scale (HADS), a self-rating scale containing two subscales measuring symptoms of anxiety (HADS-A) or depression (HADS-D). A score of >7 for either the HADS-A or HADS-D was considered abnormal, as previously recommended. Study have shown that the Cronbach’s alphas of the depression and anxiety subscales of HADS are 0.753 and 0.764 respectively, indicating good reliability and validity [19].

#### 2.2.3 Cognitive Tasks

Vigilance attention assessment was done using the psychomotor vigilance task (PVT) [20] using the computer-administered E-prime program. Subjects were instructed to press a button immediately after a stimulus (a red dot in the center of a screen on a black background) appeared. The reaction time of visual stimuli presented at random intervals of 2 seconds to 10 seconds over 10 minutes was recorded. E-prime program metrics were response speed (1/reaction time) and lapses in attention (trials >500 ms). Better vigilance attention performance was associated with faster reaction times (greater than 1/reaction time) and fewer lapses on the PVT.

Inhibitory control assessment was done using the computer-administered Stroop color-word task in the E-prime 3.0 (Psychology Software Tools, Inc., Pittsburgh, PA, USA). Inhibitory control refers to the ability of an individual to consciously suppress interference or reaction tendencies in goal-oriented activities [21]. Subjects were asked to indicate the stimulus color presented by 80 randomly ordered individual, consistent, and inconsistent stimuli. The monitoring index was the difference in reaction time between the consistent stimulus and the inconsistent stimulus when the response was correct. The smaller the difference, the better performance of inhibitory control.

Working memory was assessed using the computer-administered 2-back test. Working memory refers to temporarily storing and processing information in various complex cognitive activities [22]. Subjects were asked to judge whether the current stimulus (letter) was consistent with the second stimulus (letter). The E-prime program includes a practice part and a test part, and the index is the accuracy of the test part.

### 2.3 Procedure

**Fig. 1.**
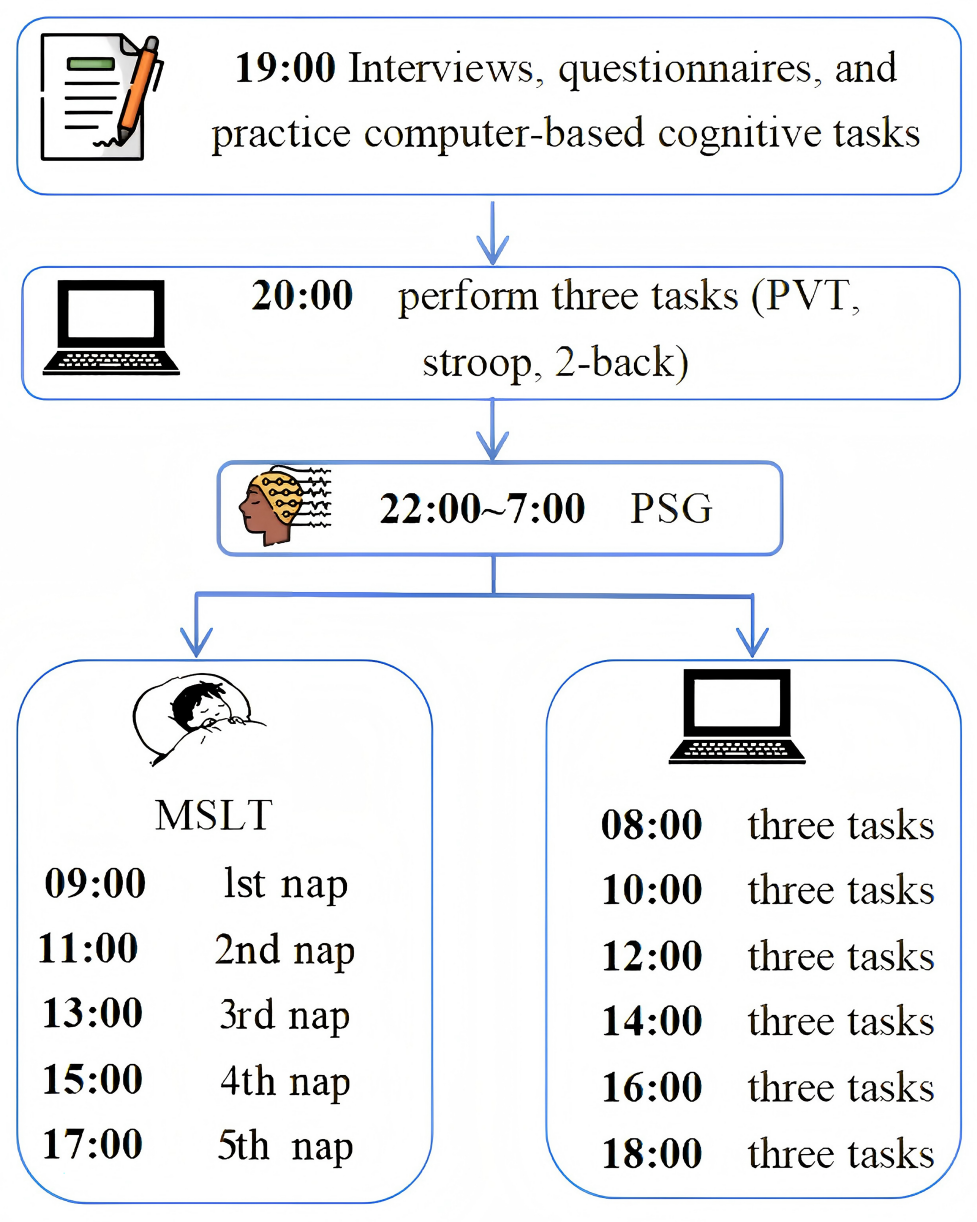
**Experimental procedure**. PVT, psychomotor vigilance task; Stroop, Stroop color-word task; 2-back, 2-back working memory task; PSG, polysomnography; MSLT, multiple sleep latency test.

When the participants of this study arrived at the Sleep Medicine Center of Nanfang Hospital, Southern Medical University, two trained researchers collected their general information through 20-minute structured interviews and guided them to complete the self-rating scales. Then, the nocturnal PSG followed by the MSLT with five naps were carried out. At the start of the nocturnal PSG as well as before and after each MSLT nap, participants completed computer-administered cognitive-behavioral tasks, including a 10-minute PVT, a 5-minute Stroop color-word task, and a 2-minute 2-back test. In addition, a pre-training procedure was set for each cognitive behavioral task before each formal test to reduce the influence of the learning effect; participants could enter the formal test only when their performance was stable. The specific experimental procedure is shown in Fig. 1.

### 2.4 Statistical Analyses

For continuous variables, the differences between groups were compared using a two-independent sample *t*-test or Mann-Whitney U test. For categorical variables, the chi-squared test was used to compare the differences between groups. The Shapiro-Wilk test was performed to check normality and Levene’s test was used to check for homogeneity of variance for continuous variables (normality: *p* > 0.05; homogeneity: *p* > 0.05). Two-factor repeated measures ANOVA was carried out with time series (different test times) as a within-subjects variable for repeated measurement and group (narcolepsy vs OSA-with-EDS) as a between-subjects factor. The main effects were compared between the narcolepsy and OSA-with-EDS groups. Non-normally distributed variables were log10-transformed to meet ANOVA assumptions. The Bonferroni test was used for post hoc comparison, and the missing values were treated with the mean. *p* < 0.05 was considered to indicate a significant difference. Data were analyzed using IBM SPSS version 25.0 (IBM Corporation, Armonk, NY, USA).

## 3. Results

### 3.1 Clinical Characteristics

Patients with narcolepsy had a significantly higher proportion of females (*p* < 0.001), a lower body mass index (BMI) (*p* < 0.001), a younger age (*p* < 0.001), markedly shorter rapid eye movement (REM) latency (*p* = 0.017), reduced percentages of N1 sleep (*p* < 0.001), increased proportions of N3 sleep (*p* < 0.001), and a significantly lower arousal index (*p* < 0.001) compared with the OSA-with-EDS group (Table 1). In addition, the mean sleep latency (MSL) during daytime was notably shorter (*p* = 0.022), accompanied by more frequent occurrences of sleep onset REM periods (SOREMP) (*p* < 0.001) in narcolepsy than in OSA-with-EDS. Furthermore, PSQI scores were significantly lower in narcolepsy compared with OSA-with-EDS (*p* = 0.003).

**Table 1.** **Demographic, questionnaire, PSG, and MSLT characteristics of the narcolepsy and OSA-with-EDS groups**.

		Narcolepsy (n = 29)	OSA-with-EDS (n = 33)	*p*-value
Sex, female, n (%)		13 (44.8%)	2 (6.1%)	<0.001
Age, years		18.93 ± 6.27	41.55 ± 12.36	<0.001
BMI, kg/m^2^		23.14 ± 4.14	27.49 ± 4.05	<0.001
Questionnaires				
	PSQI (0–21)	6.00 (4.25–8.00)	9.00 (6.75–13.25)	0.003
	MEQ-5 (0–25)	13.21 ± 3.24	13.73 ± 3.84	0.596
	HADS (0–21)	11.00 (7.25–17.25)	12.00 (5.75–17.00)	0.979
	ESS (0–24)	15.50 ± 5.64	16.03 ± 4.69	0.706
PSG				
	Total sleep time, min	430.46 ± 104.91	431.54 ± 66.95	0.961
	Sleep latency, min	5.00 (2.10–10.45)	4.50 (1.00–8.95)	0.882
	REM latency, min	72.55 (9.33–165.00)	113.20 (76.05–210.70)	0.017
	Wake after sleep onset, min	40.65 (13.39–88.90)	77.00 (31.88–130.75)	0.052
	Sleep efficiency, %	87.01 ± 12.70	82.69 ± 11.62	0.171
	REM sleep percentage, %	22.92 ± 8.20	19.79 ± 6.14	0.094
	N1 sleep percentage, %	10.20 (6.60–17.45)	23.00 (15.40–47.70)	<0.001
	N2 sleep percentage, %	45.82 ± 11.45	45.36 ± 13.63	0.887
	N3 sleep percentage, %	18.40 (13.30–24.00)	2.30 (0.00–13.90)	<0.001
	Arousal index	14.10 (10.70–17.95)	36.50 (19.70–61.00)	<0.001
	Apnea-hypopnea index	2.40 (0.45–5.05)	49.50 (20.45–71.20)	<0.001
MSLT				
	Sleep onset in REM periods, n	3.50 (2.00–5.00)	1.00 (0.00–2.00)	<0.001
	Mean sleep latency, min	2.15 (1.40–3.75)	4.90 (2.20–7.95)	0.022

BMI, body mass index; PSQI, Pittsburgh Sleep Quality Index; HADS, Hospital Anxiety and Depression Scale; ESS, Epworth Sleepiness Scale; OSA, obstructive sleep apnea; EDS, excessive daytime sleepiness; REM, rapid eye movement; MEQ-5, Morningness-Eveningness Questionaire-5.

### 3.2 Cognitive-Behavioral Results

**Fig. 2.**
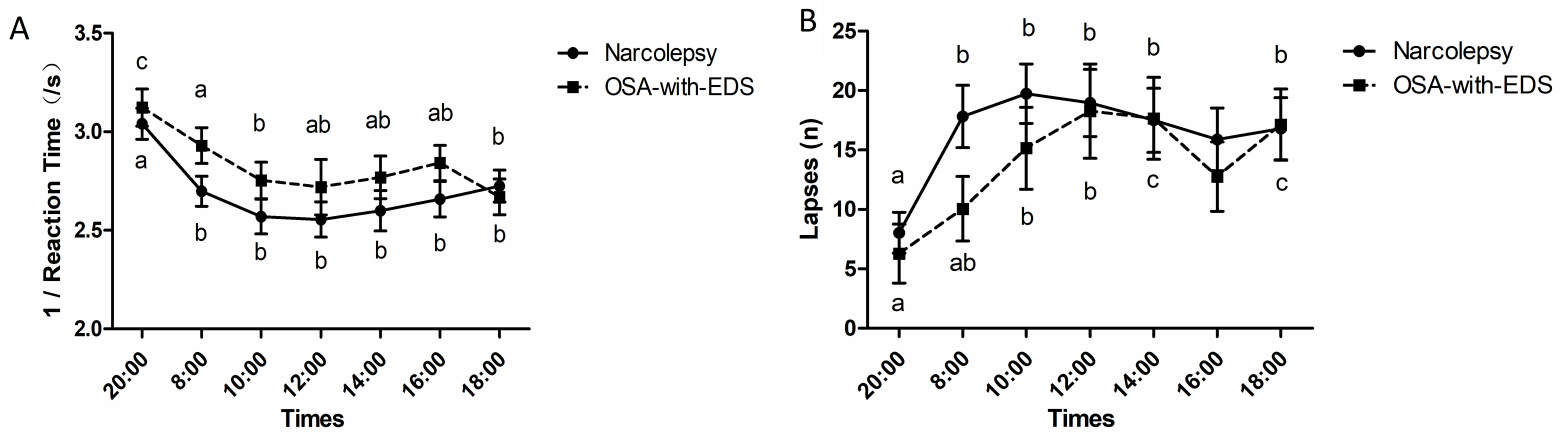
**Circadian variations of vigilant attention in the psychomotor vigilance task**. (A) Circadian variations of response speed in the psychomotor vigilance task between the narcolepsy and OSA-with-EDS groups. (B) Circadian variations of lapses in attention in the psychomotor vigilance task between the narcolepsy and OSA-with-EDS groups. Time points sharing the same letter (a, b, c) on a curve are not significantly different from each other (*p* > 0.05). Different letters denote statistically significant variations between specific time points (*p* < 0.05, repeated measures ANOVA with Bonferroni correction). Error bars represent the mean ± standard error of the mean (SEM).

A repeated measures ANOVA with a Greenhouse-Geisser correction determined that the response speed (1/reaction time) between the narcolepsy and OSA-with-EDS groups was not statistically significant (*p*_group _ = 0.248) but differed significantly between time points (*p*_time point_ < 0.001) (Table 2). The response speed of the PVT in the narcolepsy group at 20:00 was significantly greater than at other time points (*p* < 0.05). The response speed of the PVT in the OSA-with-EDS group at 20:00 was significantly greater than at other time points, and at 08:00 was significantly greater than at 10:00 and 18:00 (*p* < 0.05) (Fig. 2A).

**Table 2.** **Results of a two-factor repeated measures ANOVA between the narcolepsy and OSA-with-EDS groups**.

		20:00	08:00	10:00	12:00	14:00	16:00	18:00	*p*-value
1/reaction time of PVT	Narcolepsy (n = 29)	3.04 ± 0.43	2.70 ± 0.41	2.57 ± 0.48	2.55 ± 0.48	2.60 ± 0.55	2.66 ± 0.49	2.72 ± 0.44	*p*_group _ = 0.248
	OSA-with-EDS (n = 33)	3.12 ± 0.54	2.93 ± 0.52	2.75 ± 0.53	2.72 ± 0.81	2.77 ± 0.62	2.84 ± 0.52	2.67 ± 0.52	*p*_time point_ < 0.001
									*p*_group × time point_ = 0.089
Number of PVT lapses	Narcolepsy (n = 29)	8.03 ± 9.26	17.82 ± 14.13	19.74 ± 13.46	18.97 ± 15.19	17.50 ± 14.50	15.89 ± 14.20	16.80 ± 14.08	*p*_group _ = 0.487
	OSA-with-EDS (n = 33)	6.28 ± 14.20	10.06 ± 15.65	15.15 ± 19.79	18.27 ± 22.83	17.67 ± 19.79	12.76 ± 16.75	17.14 ± 17.20	*p*_time point_ < 0.001
									*p*_group × time point_ = 0.070
Differential reaction time of inhibition control	Narcolepsy (n = 29)	58.45 ± 48.51	44.75 ± 50.35	39.07 ± 62.60	25.59 ± 56.52	30.15 ± 58.81	35.77 ± 45.90	39.14 ± 31.71	*p*_group _ = 0.009
	OSA-with-EDS (n = 33)	77.08 ± 45.10	63.78 ± 49.54	65.40 ± 45.17	57.05 ± 49.95	57.05 ± 47.95	59.75 ± 55.40	61.85 ± 47.19	*p*_time point_ = 0.016
									*p*_group × time point_ = 0.969
2-back accuracy	Narcolepsy (n = 29)	65.00 ± 13.25	67.97 ± 14.16	71.12 ± 12.93	72.20 ± 15.56	72.32 ± 16.15	74.11 ± 16.40	75.99 ± 12.93	*p*_group _ = 0.308
	OSA-with-EDS (n = 33)	59.78 ± 13.71	61.34 ± 17.02	66.33 ± 18.83	70.22 ± 17.42	70.46 ± 16.42	72.33 ± 17.30	72.27 ± 14.41	*p*_time point_ < 0.001
									*p*_group × time point _= 0.462

The number of PVT lapses in attention was not statistically significant between the two groups (*p*_group _ = 0.487), while with a Greenhouse-Geisser correction, a notable time series effect was present (*p*_time point_ < 0.001) (Table 2). Further comparisons showed that the number of PVT lapses in the narcolepsy group at 20:00 was markedly less than at 08:00, 10:00, 12:00, 14:00, and 18:00 (*p* < 0.05). The number of PVT lapses in the OSA-with-EDS group at 20:00 was significantly less than at 10:00, 12:00, 14:00, and 18:00, and at 08:00 was significantly less than at 14:00 and 18:00 (*p* < 0.05) (Fig. 2B).

**Fig. 3.**
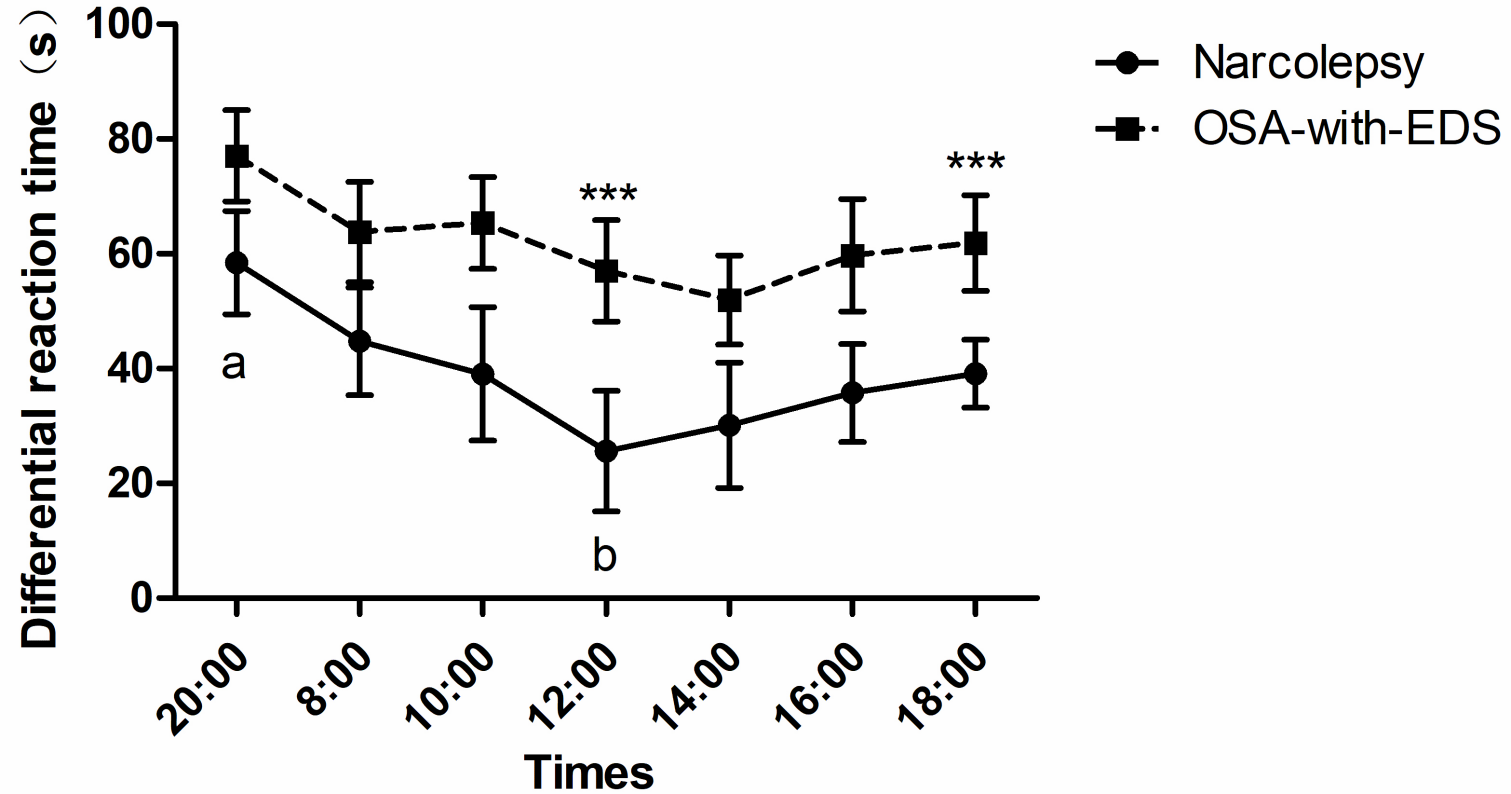
**Circadian variations of inhibitory control in the Stroop color-word task**. Time points with statistically significant differences between groups are marked with asterisks (***). Time points labeled with different letters (a, b) on the same curve are statistically different (*p* < 0.05). Error bars represent the mean ± SEM.

The differential reaction time of inhibition control between the narcolepsy and OSA-with-EDS groups displayed marked group effects (*p*_group _ = 0.009), and post hoc comparisons showed that the differences between the two groups were significant at 12:00 (*p* = 0.025) and 18:00 (*p* = 0.033). ANOVA with a Greenhouse-Geisser correction showed a significant difference in time points (*p*_time point_ = 0.016) (Table 2). Additionally, an increased difference in reaction time was seen among the narcolepsy group at 20:00 relative to 12:00 (*p* = 0.049), but no difference in differential reaction time was seen in the OSA-with-EDS group (Fig. 3).

**Fig. 4.**
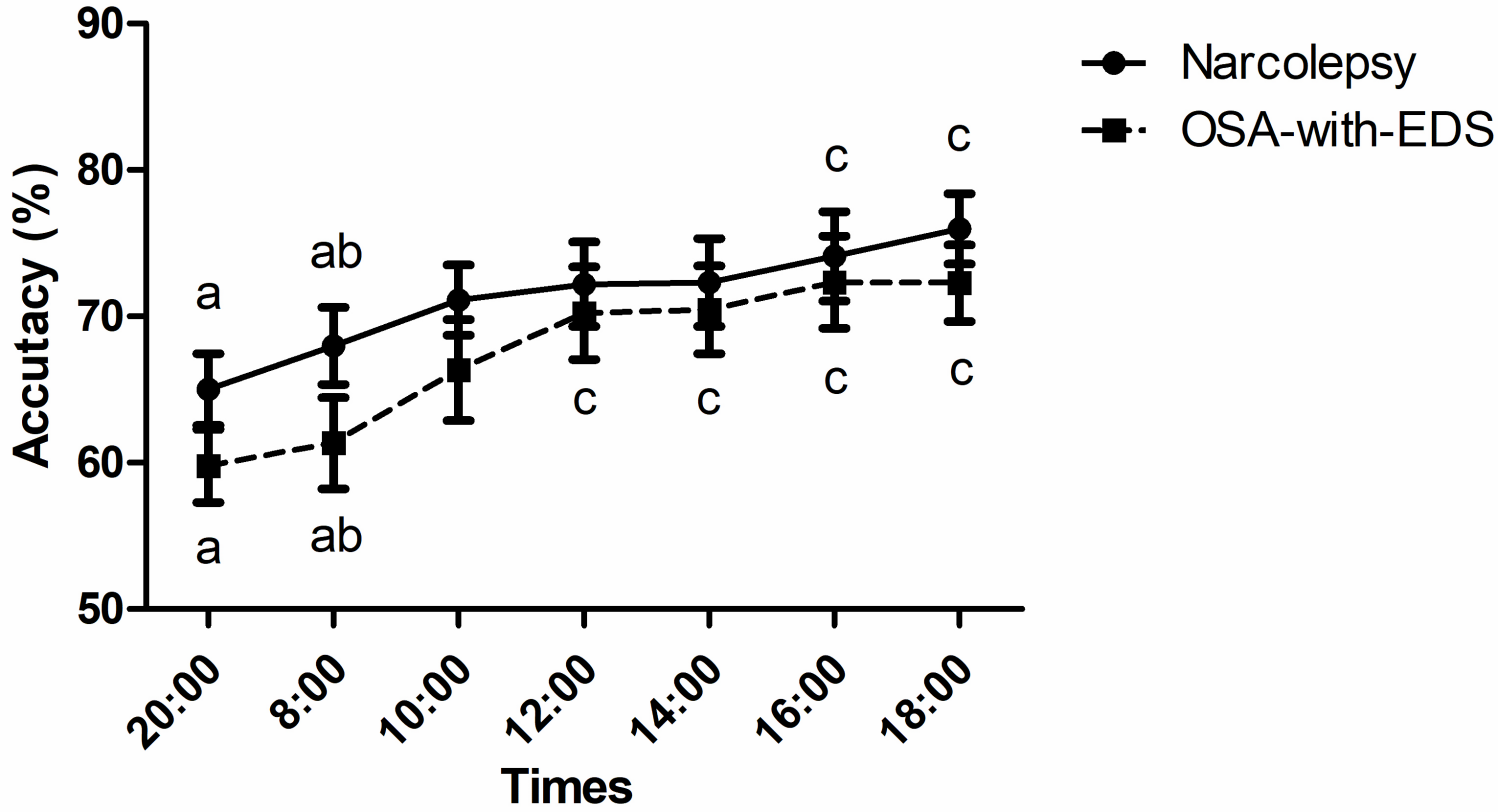
**Circadian variations of 2-back working memory**. Time points sharing the same letter (a, b, c) on a curve are not significantly different from each other (*p* > 0.05). Different letters denote statistically significant variations between specific time points (*p* < 0.05, repeated measures ANOVA with Bonferroni correction). Error bars represent the mean ± SEM.

The 2-back accuracy between the narcolepsy and OSA-with-EDS group had no statistically significant between-group differences (*p*_group _ = 0.308), however, the Greenhouse-Geisser corrected time series effect was significant (*p*_time point_ < 0.001) (Table 2). Post-hoc tests showed that the accuracy in the narcolepsy group at 16:00 and 18:00 was significantly higher than at 20:00 and 08:00 (*p* < 0.05). The accuracy in the OSA-with-EDS group at 12:00, 14:00, 16:00, and 18:00 was significantly higher than at 20:00 and 08:00 (*p* < 0.05) (Fig. 4).

## 4. Discussion

Our study revealed that patients with narcolepsy exhibited significant circadian variations in vigilant attention, inhibitory control, and working memory. The circadian variations of cognitive performance in the narcolepsy group showed similar trends in diurnal variation as those of patients with OSA-with-EDS. However, patients with narcolepsy performed poorly in vigilant attention tasks but performed better in inhibitory control and working memory tasks.

Narcolepsy patients showed the lowest vigilant attention performance at noon followed by recovery and the best vigilant attention performance was seen in the evening. A previous study revealed similar results, in that sustained attention showed fluctuations in performance in patients with narcolepsy, with declines in the morning and at noon [23]. Other studies aimed at vigilant attention in patients with EDS performed poorly at 07:00 [24], even lower at 13:00, and improved by 18:00 [25]. Research has also indicated that the attention of normal individuals shows relatively consistent circadian variability compared with those of patients with narcolepsy. It has been reported that attention is relatively low in the morning, improves throughout the midday period, experiences a decline post-lunch, and subsequently improves during the afternoon and early evening hours [8, 26]. Furthermore, as for vigilant attention between different groups, studies have found that patients with narcolepsy performed worse compared with healthy controls [9, 27]. In summary, the circadian variations of vigilance attention in narcolepsy show similar time trends in diurnal variation implying similar regulatory mechanisms for the circadian rhythm of cognitive performance.

The inhibitory control of patients with narcolepsy showed a pattern of improvement in the morning and a decline in the afternoon, which is consistent with previous studies. A study aimed at narcolepsy patients indicated that inhibitory control was at its lowest 1–2 hours after waking [10]. A previous functional magnetic resonance imaging study found that inhibitory control activation and neural activity in associated brain regions exhibited time-dependent changes [28]. Importantly, evidence suggests that inhibitory control performance is associated with the 24-hour activity rhythm [29], and the circadian rhythm plays a fundamental role in regulating inhibitory control [10]. Moreover, a study comparing narcoleptics with healthy controls demonstrated that narcolepsy was associated with attenuated medial prefrontal cortex (mPFC) responses during inhibitory control [11]. Preclinical research has also shed light on how this impaired cognition may be a consequence of reduced hypocretin signaling in the mPFC systems [30]. In the present study there was a between-group difference in the inhibitory control task, where narcolepsy subjects performed better than the OSA-with-EDS group. This result necessitates replication prior to drawing any conclusions about the significant differences in task performance between the two groups.

The working memory of patients with narcolepsy demonstrated a gradual improvement throughout the day. A previous neuroimaging study has consistently monitored participants at multiple time series during the 2-back task, revealing that activation of the cortical executive network is diminished in individuals with narcolepsy [31], which may account for the observed changes in working memory performance in this study. Furthermore, one study reported that while attention performance was poorer in narcolepsy patients compared with controls, their memory may not be objectively impaired [32]. Conversely, another investigation identified significant group differences in procedural memory among patients with narcolepsy compared with controls [33]. This fact that the intercept was not significantly different for the 2-back task strongly suggests that group differences are not so pervasive that they can be reliably assessed by a single test run of a performance test. This may explain to some extent why results from earlier studies with working memory performance measures in narcolepsy patients were inconsistent or contradictory.

Generally, cognitive impairment of narcolepsy is assumed to be a consequence of EDS [4]. It is presumed that vigilance levels associated with EDS contribute to impairments in higher-order cognitive functions [34]. Another hypothesis posits that cognitive impairment in narcolepsy may also involve disease-specific components [35], such as the depletion of hypocretin neurons in various brain regions, including the mPFC and hippocampus [36, 37]. Our study demonstrated that patients with narcolepsy exhibited poorer vigilant attention compared with OSA-with-EDS patients while showing significantly better performance in higher-order cognitive tasks (e.g., inhibitory control). Therefore, we propose that disease specificity is an integral factor contributing to cognitive impairment.

This study has the following limitations. Firstly, the sample size of this study is relatively small, thereby potentially restricting the statistical power and augmenting the risk of incidental findings. Furthermore, owing to the constraint of the sample size, we were incapable of fully adjusting for the potential confounding effects of baseline variables in the analysis, which might have resulted in a bias in the results estimation. Further verification of the robustness of the results of this study, through larger-scale studies, is required in the future. Finally, although we screened the participants, the medication history and comorbidities of the participants were not recorded in detail, which could have influenced the final results.

## 5. Conclusions

Circadian variations are evident in cognitive performance among patients with narcolepsy across essential cognitive functions, including vigilant attention, inhibitory control, and working memory. Furthermore, when compared with the OSA-with-EDS group, intrinsic disease specificity may play an important role in the cognitive impairments associated with narcolepsy. The results of this study provide a scientific basis for optimizing the scheduling of academic and occupational activities for patients.

## Data Availability

The datasets used and analyzed during the current study are available from the corresponding authors on reasonable request.

## Abbreviations

EDS, excessive daytime sleepiness; OSA, obstructive sleep apnea; PSG, polysomnography; MSLT, Multiple Sleep Latency Test; PVT, psychomotor vigilance task; ICSD-3, International Classification of Sleep Disorders, Third Edition; AHI, apnea/hypopnea index; ESS, Epworth Sleepiness Scale; AASM, American Academy of Sleep Medicine; PSQI, Pittsburgh Sleep Quality Index; MEQ-5, Morningness-Eveningness Questionnaire-5; HADS, Hospital Anxiety and Depression Scale; BMI, body mass index; REM, rapid eye movement; MSL, mean sleep latency; SOREMP, sleep onset rapid eye movement period; mPFC, medial prefrontal cortex.
